# Lamivudine-Induced Liver Injury

**DOI:** 10.3889/oamjms.2015.110

**Published:** 2015-10-16

**Authors:** Lamidi W. B. Olaniyan, Emmanuel N. Maduagwu, Olalekan Wasiu Akintunde, Oladimeji O. Oluwayelu, Bartholomew I. C. Brai

**Affiliations:** 1 *Biochemistry Department, Faculty of Basic Medical Sciences, Ladoke Akintola University of Technology, Ogbomoso, Nigeria*; 2 *Biochemistry Department, College of Medicine, University of Ibadan, Ibadan, Nigeria*; 3 *Anatomy Department, Faculty of Basic Medical Sciences, Ladoke Akintola University of Technology Ogbomoso, Ogbomoso, Nigeria*; 4 *Veterinary Microbiology and Parasitology Department, Faculty of Veterinary Medicine, University of Ibadan, Ibadan, Nigeria*; 5 *Molecular Biology and Biotechnology Division, Nigerian Institute of Medical Research Lagos, Lagos, Nigeria*

**Keywords:** Embryonated egg, Histopathology, Lamivudine cytotoxicity, Oxidative, stress

## Abstract

**BACKGROUND:**

Lamivudine is a nucleoside analogue antiretroviral drug, known for its low toxicity at clinically prescribed dose. However, the toxicity or mechanism of toxicity and target tissue effects during prolonged administration of higher doses were hardly given sufficient laboratory attention.

**AIM:**

The present work was designed to investigate the biochemical and histopathological changes in the liver of rat administered with prolonged doses of lamivudine.

**MATERIAL AND METHODS:**

Lamivudine in multiple doses of five ranging from 4 mg/kg to 2500 mg/kg were administered, in vitro, by injection into the air-sac of 10–day old fertile embryonated eggs of Gallus domesticus. Also, female rats of the Wistar strain received oral doses, up to 500 mg/kg singly or repeatedly for 15 or 45 days, respectively. Spectrophotometric techniques were employed to monitor activities of the aminotransferases (ALT and AST), γ–glutamyltransferase (GGT) and total protein concentration in serum while activities of glutathione S–transferase (GST), GGT and superoxide dismutase (SOD) as well as concentrations of malondialdehyde (MDA) and protein were determined in liver. Histopathological studies were carried out on liver. Data were analysed using ANOVA and were considered significant when p < 0.05.

**RESULTS:**

The LD50 for the drug calculated from the incubation experiment was 427 mg/kg. Total serum protein concentration significantly reduced while enzymes activities significantly increased at 500 mg/kg only among the repeat-dosed rats. Hepatic GGT, GST and SOD activities as well as MDA concentration were significantly elevated at 20 mg/kg. Histopathological studies showed multifocal lymphoid cell population in the liver sinusoid of the chicken and hydropic degeneration of hepatocytes were recorded among rats repeatedly exposed to the drug respectively at doses ≥ 100 mg/kg.

**CONCLUSION:**

Lamivudine toxicity in rat liver appeared to be mediated by oxidative stress.

## Introduction

Lamivudine is a cytidine - analogue antiretroviral pro-drug. It is metabolically activated into 51-triphosphorylated derivative by cytosolic kinases in a step-wise manner in the liver. The actively metabolised drug inhibits polymerase gamma, the enzyme that catalyses the synthesis of mitochondrial DNA which may lead to mitochondrial dysfunction in the susceptible tissue [1] with the accompanied clinical effects. Oxidative stress, an imbalance between prooxidant and antioxidant levels favouring the former may arise profoundly as a result of mitochondrial dysfunction, underlies mechanism of a number of drug toxicities [2, 3].

Lamivudine is largely excreted in the urine unchanged or to a minor extent as a trans-sulphoxide [4]. Although the drug has been reported to have limited toxicity relative to its pharmacologic counterparts [5], pancreatitis [6], polyneuropathy [7] and Parkinsonism [8] have been documented in patients on prolonged therapy.

Generally, much of the toxicologic information about this drug were obtained from clinical experience and the drug effects on liver especially following prolonged therapy at high doses, had not been precisely determined.

Therefore, the objective of this work was to establish toxic effects of the drug on rat liver at supra-therapeutic doses and specifically the mechanism of the toxicity.

## Materials and Methods

Standard solutions of the drug (Lamivir brand containing 150 mg lamivudine) were prepared in normal saline or in distilled water.

### Embryonic in vitro study

This was carried out according to the method of Gabliks et al., [9]. An aliquot of 0.5 ml of the drug in normal saline equivalent to 4, 20, 100, 500 or 2,500 mg/kg was injected into the chorioallantoic sac of 10-day old fertile embryonated egg (average weight 55 g) of Gallus domesticus, using 30 eggs per dose level. An aliquot of 0.5 ml normal saline only, injected into each embryonated egg of equal number served as the control. The response dose (RD50) was calculated from the data obtained using the normograph of Tint and Gillen [10].

### Animal experiments

Four dose levels, 4, 20, 100, and 500 mg/kg [11, 12], in maximum volume of 2ml. normal saline were adopted. The start dose was the recommended therapeutic dose for humans. The drug was administered orally in single doses to the adult female rats of Wistar strain. There were six rats per dose level. The control rats of equal number received 2ml. of normal saline only. Another group of rats on similar dose regimens were then placed on repeated (daily) oral administration. Both animal groups were observed for 14 and 45 days respectively in individual plastic animal cages. All the animals had access to normal chow and potable water ad libitum.

### Sample preparations

Serum samples were prepared from the rat blood and stored in vacutainers. Livers were carefully removed rapidly, washed off blood as well as connective tissues with 1.15% ice-cold KCl solution. Homogenization of the sample took place in ice-cold 0.25 M sucrose solution buffered at pH 7.4 with 40 mM Tris. HCl with a Potter Elvherjem homogenizer. Each sample homogenate was centrifuged at 8000g for 10 minutes in 20% (w/v) buffered medium. The pellet so obtained was taken up in 10ml. of the medium and re-centrifuged. The supernatants were centrifuged at 12000g for 10 minutes to remove light mitochondria [13]. The final supernatants were combined and used as post-mitochondrial fraction for the biochemical analyses.

### Analytical methods

In the sera, aspartate and alanine aminotransferases activities were determined using RANDOX (UK) Laboratories analytical kits while total protein concentrations were determined according to Lowry et al., [14], and malondialdehyde (MDA) concentration according to Yuda et al. [15]. Activity of glutathione S- transferase was measured using the method of Habig et al. [16], that of γ- glutamyltransferase (GGT) was by [17] while that of superoxide dismutase (SOD) was by [18], respectively in the hepatic post-mitochondrial fractions.

### Histopathology

The harvested liver tissues of both the chicks and rats were fixed in formol saline for 24 hours and dehydrated thereafter using ethanol in ascending concentrations starting from 70%. The tissues were cleared using xylene and infiltrated with melted wax followed by embedding, sectioning, rehydration in descending grades of ethanol in that order and finally staining with hematoxylene and eosin. Histopathological studies were carried out on the prepared slides and examined under light microscope.

### Statistical analyses

One-way analysis of variance (ANOVA) with Dunnett multiple comparisons unpaired t-test was employed. Data were considered significant when p<0.05.

## Results and Discussion

The response dose (RD_50_), the drug dose that killed/caused unhatching of half the population of the chick embryos was calculated as 427 mg/kg (data not shown). No drug-induced mortality among the rats was recorded. Body-weight gain was comparatively reduced in rats on repeated administrations (Tables 1) which might be drug - related (r = - 0.822).

**Table 1:** Body-weight change of rats following repeated daily oral doses of lamivudine for 45 days*

Lamivudine dose (mg/Kg/day)	Initial weight (g)	Final weight (g)	Weight change (%)
0.0	158.7 ± 2.62	208.2 ± 4.74	+31.2 ± 1.14
4.0	140.4 ± 2.45	177.7 ± 6.04†	+28.0 ± 1.14
20.0	117.0 ± 4.04	142.4 ± 7.14†	+21.3 ± 2.12
100.0	163.4 ± 3.80	197.9 ± 6.41†	+21.0 ± 1.25
500.0	150.1 ± 4.78	172.1 ± 7.31†	+14.5 ± 1.47

* Values are means ± S.E.M.

† Means significantly different when compared with the control P<0.05 otherwise p>0.05 (Paired t- test).

There were no statistically significant changes in the activities of the serum enzymes as well as protein concentration in rats dosed with single oral administrations when compared with the control or with 4 mg/kg (data not shown). However, the increased serum enzymes activities accompanied with reduced serum protein concentration in rats repeatedly exposed to the drug were statistically significant at 500 mg/kg only, when compared with the control (Table 2).

**Table 2:** Sera of rats following daily oral doses of 3TC for 45 days*

Lamivudine dose (mg/kg/d)	ALT (I.U/L)	AST (I.U/L)	GGT (I.U/L)	Total Protein (mg/ml)
0.0	23.3087 ±1.51	42.5710 ± 1.96	14.3075±0.72	6.134 ± 0.61
4.0	25.0575 ± 1.31	43.1957 ± 1.18	13.8633 ± 0.81	6.186 ± 0.35
20.0	27.7433 ± 1.99	43.4660 ± 1.38	15.2150 ± 1.12	5.325 ± 0.34
100.0	28.4802 ± 2.53	44.7498 ± 2.34	15.8780 ± 2.65	4.791 ± 0.45
500.0	33.1847 ± 3.96†	56.4205±7.17†	16.2613 ± 1.78†	3.945 ± 0.20‡

* Values are means ± S.E.M. Mean significantly different from the control

† p<0.05;

‡ p<0.01, otherwise p>0.05.

The specific activities of hepatic GST and GGT in rats were found to increase with applied doses (Table 3). The specific activities of both enzymes were significantly increased by as little a dose as 20 mg/kg when compared with the control.

**Table 3:** Specific activities of GST and GGT in rat livers following daily oral doses of 3TC for 45days^#^

Lamivudine-doses (mg/kg/day)	GST specific activity (U x 10^-2^/mg protein)	GGT specific activity (U/mg protein)
0.0	8.7833 ± 0.75	3.5487 ± 0.51
4.0	11.4250 ± 1.43	6.1883 ± 1.16
20.0	14.9767 ± 1.71^†^	10.2442 ± 1.97^†^
100.0	15.8083 ± 1.71^†^	12.4675 ± 1.17^*∆^
500.0	18.2333 ± 2.09^*∆^	15.3658 ± 2.24^*‡^

# Values are means ± S.E.M. Mean significantly different from the control

† p<0.05,

* p<0.01, and from 4mg/kg,

∆ p<0.05,

‡ p<0.01, otherwise p>0.05 when compared with the control.

Also, the hepatic concentration of MDA and specific activity SOD increased in a similar fashion as GST and GGT (Table 4). In comparison with the control, the increased concentration of hepatic MDA was significant as from 100 mg/kg whereas the mean specific activity of SOD was significantly increased by the 20 mg/kg dose (Table 4).

**Table 4:** Malondialdehyde (MDA) concentrations and superoxide dismutase (SOD) specific activities in the rat livers following daily oral doses of 3TC for 45 days^#^

Lamivudinedose (mg/kg/day)	Hepatic-MDA concentration (nmole/mg protein)	Hepatic SOD specific activity-(U/mg protein)
0.0	0.0571 ± 0.01	9.9583 ± 0.70
4.0	0.0730 ± 0.01	12.0100 ± 0.93
20.0	0.1560 ± 0.03	14.7017 ± 1.49^*^
100.0	0.1647 ± 0.03^†^	14.7383 ± 1.20^*^
500.0	0.2228 ± 0.04* ‡	16.5267 ± 1.32^*∆^

# Values are means ± S.E.M. Mean significantly different from the control

† p<0.05,

* p<0.01 and different from 4mg/kg,

∆ p<0.05, ‡p<0.01, otherwise p>0.05 when compared with the control.

The mean ratio of the liver-weight to the total body-weight among the rats repeatedly exposed to the drug increased with the dose. The effect of 500 mg/kg was very significant (Table 5).

**Table 5:** Liver-weight/Body-weight change in the rats following daily oral doses of 3TC for 45 days^#^

Lamivudine dose (mg/kg/day)	Liver/Body weight ratio (x 10^-5^)
0.0	3.4683 ± 0.051
4.0	3.4950 ± 0.077
20.0	3.5900 ± 0.065
100.0	3.6200 ± 0.055
500.0	4.0067 ± 0.102^*‡^

# Values are means ± S.E.M. Mean significantly different, from the control

* p<0.01, from 4mg/kg

^∆^p<0.05, p<0.01 otherwise p>0.05 (not significant) when compared with the control.

**Figure 1:**
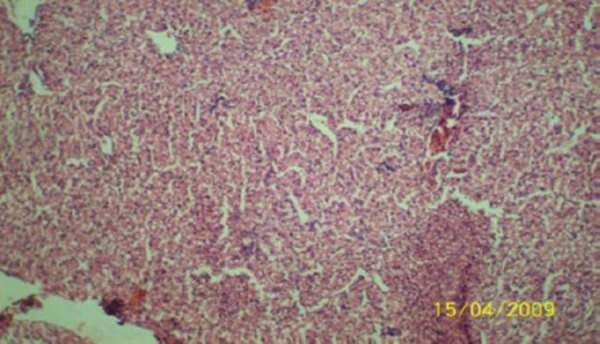
*Liver (H & E ×100) of chick embryo following injection of 0.5 ml normal saline (the control) showing no visible lesion*.

**Figure 2:**
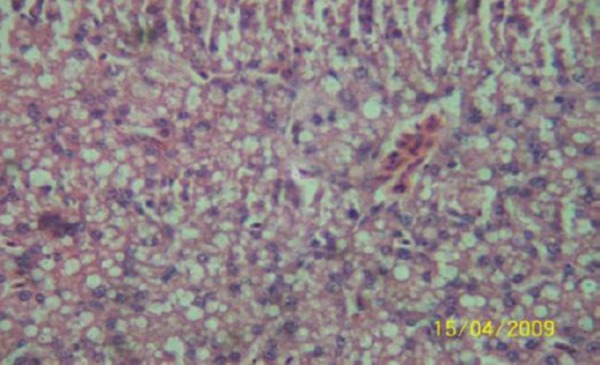
*Liver (H & E ×100) of the chick embryo following injection of 4 mg 3TC/kg body weight showing lymphoid aggregates, very sparse*.

**Figure 3:**
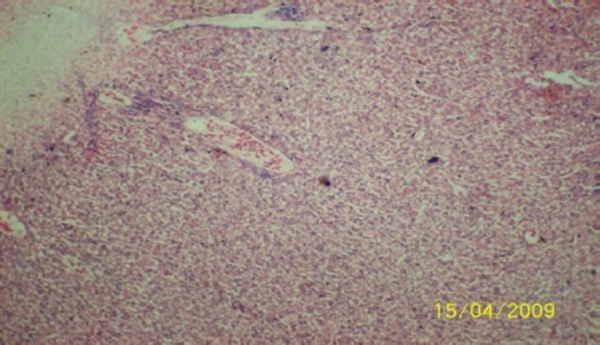
*Liver (H&E ×100) of the chick embryo following injection of 20 mg 3TC/kg body weight showing lymphoid aggregates very sparse and very mild portal congestion*.

**Figure 4:**
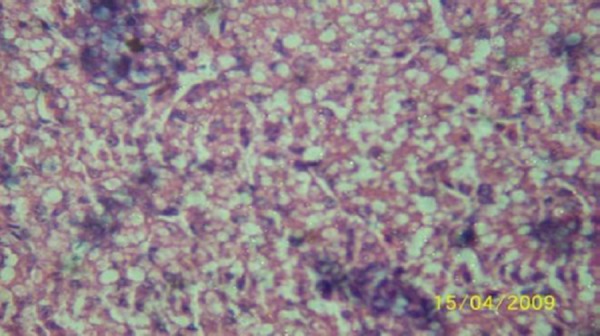
*Liver (H & E × 100) of chick embryo following injection of 100 mg 3TC/kg body weight showing moderately dense lymphoid compartment*.

**Figure 5:**
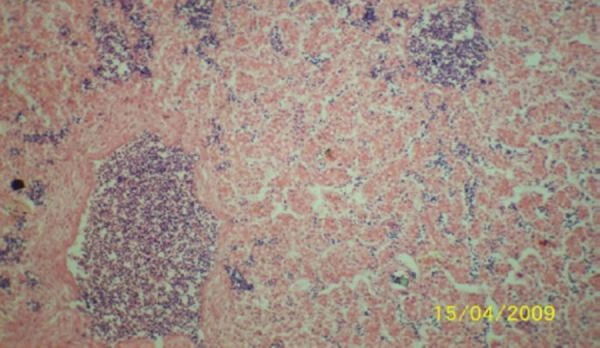
*Liver (H & E × 100) of chick embryo following injection of 500 mg 3TC/kg body weight showing highly dense multifocal lymphoid cell population in the sinusoid*.

**Figure 6:**
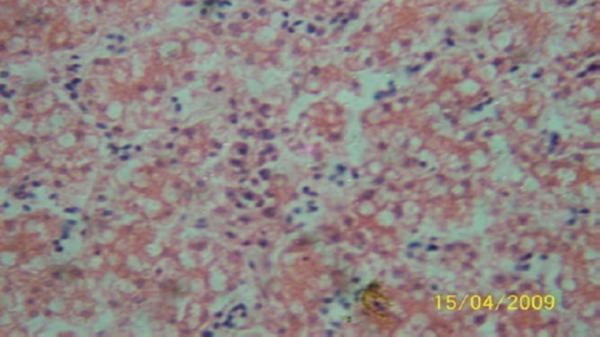
*Liver (H & E x 100) of chick embryo following injection of 2500 mg 3TC/kg body weight showing highly dense multifocal lymphoid cell population in the sinusoid*.

**Figure 7:**
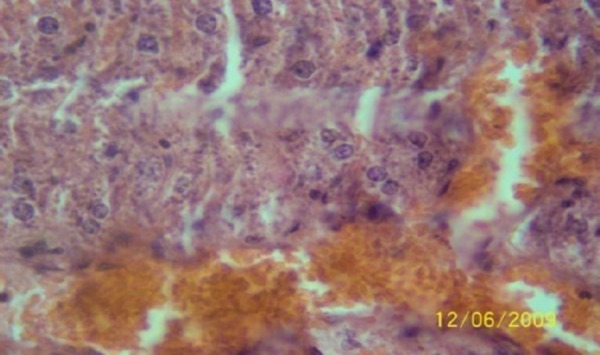
*Liver (H & E ×400) of the rat administered orally 0.2 ml of distilled water daily for 45days (the control) showing no visible lesion*.

**Figure 8:**
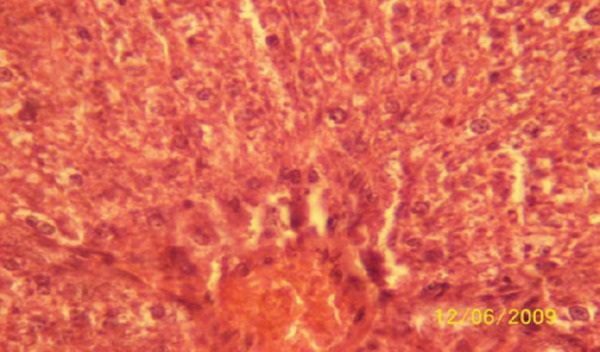
*Liver (H & E × 400) of the rat dosed orally 4 mg 3TC/kg body weight daily for 45days showing no visible lesion*.

**Figure 9:**
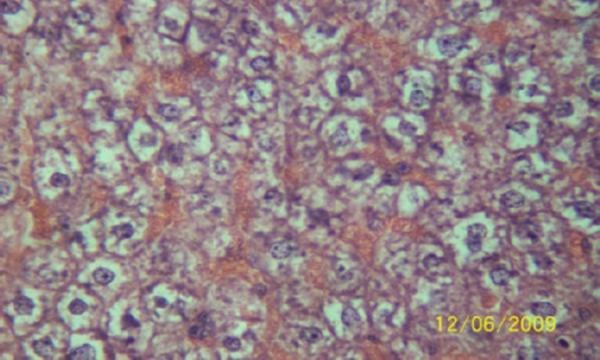
*Liver (H & E ×400) of the rat dosed orally 20 mg 3TC/kg body weight daily for 45 days showing marked sinusoidal and portal congestion with diffuse hydropic degeneration of hepatocytes*.

**Figure 10:**
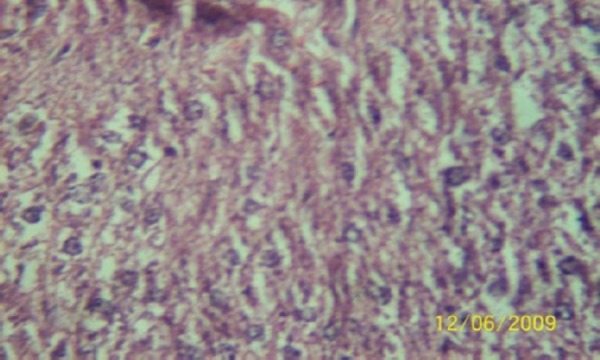
*Liver (H & E ×400) of the rat following daily oral dose of 100 mg 3TC/kg body weight for 45 days showing severe diffuse hydropic degeneration of hepatocytes*.

**Figure 11:**
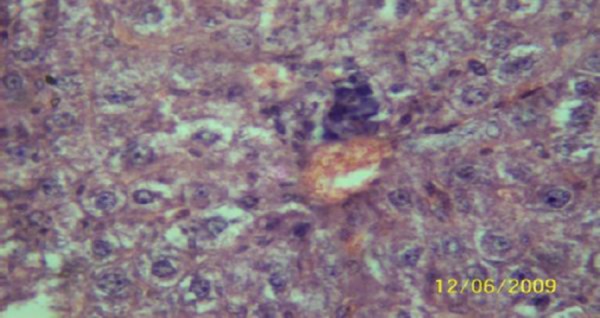
*Liver (H & E ×400) of the rat dosed 500 mg 3TC/kg body weight daily for 45days showing marked sinusoidal and portal congestion with severe hydropic degeneration of hepatocytes. There is also cellular infiltration (periportal) by macrophage*.

The results of the histopathologic investigations of the liver tissues of both the chick embryos and the rats following the administration of lamivudine are presented in Figures 1 to 6 and 7 to 11 respectively. It was revealed that as little a dose as 4 mg/kg, (the human therapeutic equivalent of the drug), produced lymphoid aggregates in the chick embryo livers but 20 mg/kg dose produced similar effects in the rats exposed repeatedly to the drug.

The fertile avian embryo is highly sensitive and susceptible to the toxicity of xenobiotics. In addition, the presence of phases 1 and 2 drug-metabolising enzymes in the chicken embryo [19, 20] informed our use of the egg titration/embryonation technique for our *in vitro* toxicity studies. The LD_50_ 427 mg/kg, the dose responsible for the death of half the population of the embryos, informed our choice of 500 mg/kg as the terminal dose in the animal experiments, and the dose differential in multiples of five as applied is in accord with the geometric progression requirement of moving average method of LD_50_ determination [11].

The observed reduction in body-weight gain among the rats on repeated doses relative to the control or to those on single doses could be associated with the drug [21]. Body-weight is known to be determined by complex mechanisms regulating energy balance. A number of neurotransmitter systems acting in several hypothalamic nuclei are pivotal to the regulation of body fat stores. Reduced adipose tissue has been foremost cause of low body-weight [22]. Abnormal fat metabolism or lipodystrophy has been reported as a consequence of mitochondrial dysfunction associated with many of the nucleoside/nucleotide reverse transcriptase inhibitors [23].

The increased activities of serum enzymes resulted from their release into blood circulation following structural damage to the liver cells by the drug 24]. The low levels of total protein in sera of these animals reflected negative protein turn-over which occurs in pathologic livers [25]. The results of the serum analyses in rats on single doses seemed to show animal recovery from the boost of the drug doses [26].

GGT and GST, both GSH-dependent enzymes, are found in large amounts in the liver. Their respective increases in mean specific activities sustained by GSH supply, was probably an adaptive response to the oxidative challenge occasioned by the drug.

MDA is a product of lipid peroxidation. Lipid peroxidation is a widely accepted mechanism of cellular injury and death. The elevated MDA levels in the rat livers suggested onset of oxidative stress presumably by GSH depletion [27]. The increased specific activity of SOD concerned with superoxide dismutation, might be an adaptive response to the sustained oxidative challenge by the drug.

The observed increase in liver- weight/body-weight ratio could be connected with lipodystrophy, abnormal fat metabolism; a clinical presentation in NRTI- exposed patients, associated with mitochondrial dysfunction [27]. Further work is needed to confirm lipodystrophy in lamivudine – exposed subject whereas data here presented could be a seminal contribution towards quantitative proof of lamivudine-induced lipodystrophy.

In conclusion, the result of the investigations suggested hepatotoxic potentials of lamivudine at high doses (≥ 100 mg/kg) on prolonged administration. Oxidative stress appeared to be the drug mechanism of the toxicity in rat liver. According to the reported British Toxicological Society rating [28], 500 mg 3TC/kg repeatedly taken orally for such periods of time, was ‘harmful’ to the female *Wistar* rat.
